# Abdominal changes in patients with degenerative spinal deformity: A comparative study

**DOI:** 10.1097/MD.0000000000026851

**Published:** 2021-10-01

**Authors:** Chen Guo, Shuai Xu, Yan Liang, Fanqi Meng, Zhenqi Zhu, Haiying Liu

**Affiliations:** Department of Spinal Surgery, Peking University People's Hospital, Peking University, No. 11 Xizhimen South Street, Xicheng District, Beijing, P.R. China.

**Keywords:** abdominal cavity volume, clinical outcomes, degenerative kyphosis, degenerative lumbar scoliosis, degenerative spinal deformity, diaphragm rotation, lumbar stenosis syndrome, thoracolumbar kyphosis

## Abstract

The incidence of degenerative spinal deformity (DSD) is increasing with the age while the effect of DSD on the abdominal cavity parameters is unclear.

To identify the characteristics of abdominal change in DSD and to explore the correlation between the abdominal cavity volume (ACV) and various types of DSD.

The retrospective study included 95 patients with DSD and 100 subjects without deformity as control group. The Cobb angle, thoracic kyphosis angle, thoracolumbar kyphosis (TLK) angle, and lumbar kyphosis angle were obtained through full-length X-ray. The ACV was calculated by measuring the longitudinal, transversal, and coronal diameters of the abdominal cavity on magnetic resonance imaging (MRI). The rotation of the diaphragm (DR) were measured in the sagittal plane. DSD ones were divided into degenerative lumbar scoliosis (DLS group), degenerative kyphosis (DK group), and degenerative lumbar scoliokyphosis (DKS group).

Compared to control group, ACV of the DLS and DKS group was smaller. The distance between the xiphoid process and spine in DLS group was shorter and DR in DK group and DKS group was smaller. The inter-group analysis showed ACV and the shortest distance between xiphoid process and spine in DLS and DKS group were significantly lower than those in DK group. The degree of DR in DK group and DKS group was higher than that in the DLS group. Multiple linear regression analysis showed Cobb angle and weight were influencing factors of ACV with ACV = 0.67 × weight – 0.19 × Cobb angle + 2231.8. The DR was affected by TLK with DR = 25.82 – 0.42 × TLK.

DLS can cause the decrease of ACV. DK will not cause changes of ACV but is related to the degree of kyphosis. DKS will impact both ACV and DR.

## 1 Introduction

Degenerative spinal deformity (DSD) refers to sagittal or coronal malformations of the lumbar or thoracolumbar spine caused by degeneration in adulthood, affecting 32% to 68% of population over 65 years old.^[1]^ Bess et al^[2]^ believed that degenerative lumbar scoliosis (DLS) had a series of systemic adverse, which might compress the abdominal organs,^[3]^ resulting in abdominal complications and even respiratory function deterioration.^[4]^ Ailon et al^[5]^ believed that kyphosis may negatively impact on health including decreased physical function, restricted pulmonary function and poor quality of life. However, there were still seldom researches and evidence on mechanism of organs dysfunction influenced by DSD.

Radiological parameters on organs have been widely proved to be correlated to their function. It has been confirmed that thoracolumbar kyphosis (TLK) caused by ankylosing spondylitis (AS) can seriously affect the digestive and pulmonary function,^[6]^ which was further identified that the digestive dysfunction derived from the decrease of abdominal cavity volume (ACV) while respiration downregulation was attributed to the decrease of thorax elasticity and sagittal rotation of diaphragm. Thus, orthopedics not only restored the spinal sequence but also reduced the compression of abdominal organs and diaphragm.^[7]^ However, there has been no quantitative radiological investigation on the change of ACV and the deformity in DSD.

Therefore, a radiographic study was performed in patients with DSD, to identify the characteristics of abdominal change in DSD and to explore the correlation between the abdominal parameters and various types of DSD in this series.

## 2 Materials and methods

### 2.1 Patient enrollment

This retrospective single-center study was approved by the ethical committee of our institution. The patients with DSD combined with lumbar stenosis syndrome (LSS) were enrolled (DSD group) from January 2015 to June 2019. With 1:1 matching according to propensity score matching methods, the LSS subjects without spine deformity well-matched to DSD group in demographics were included as control group. Patients with DSD were divided into 3 subgroups according to the deformity types: with pure DLS group, with pure degenerative kyphosis (DK group), and with kyphoscoliosis (DKS group). All participants have signed informed consents.

Criteria for selecting the subjects were as follows: The patients are older than 50 year old. Scoliosis was defined as Cobb angle greater than 10° caused by degeneration. Kyphosis was defined as a TLK exceeding 20° caused by degeneration.^[8]^ Intact MRI of the thoracolumbar spines were available. Stand radiographs of lumbar and the whole spine were available. All cases were with severed LSS reaching to surgical indications. The exclusion criteria were congenital or idiopathic spinal deformity; other spinal deformity such AS or Scheuermann Disease; cases without LSS or with mild LSS; infection, tumor, or fracture on spine; previous spinal surgeries; or previous abdominal or thoracic surgeries.

### 2.2 Spinal alignment evaluation

**Figure 1:**
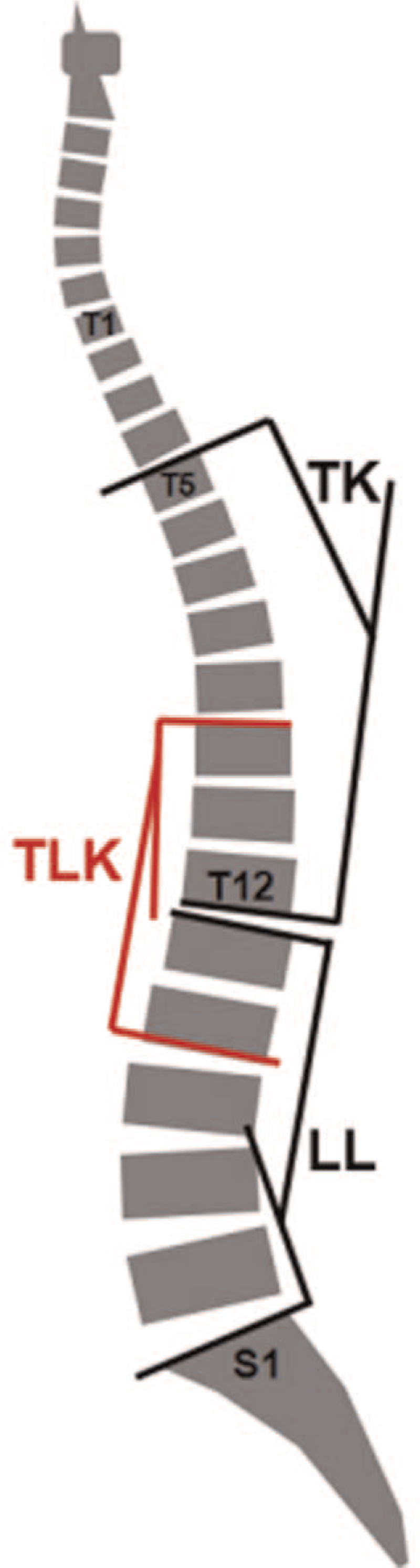
Measurements of spine alignment on neutral lateral X-ray. TK: the sagittal angle between superior endplate of T5 and inferior endplate of T10; TLK: the sagittal angle between superior endplate of T10 and inferior endplate of L2; LL: the sagittal angle between superior endplate of T12 and inferior endplate of S1. LL = lumbar lordosis, TK = thoracic kyphosis, TLK = thoracolumbar kyphosis.

The standard radiographs were undertaken using a long-cassette standing radiographs of the spine to identify: DLS, DK, or KS; Cobb angle; thoracic kyphosis (the sagittal angle between superior endplate of T5 and inferior endplate of T10, which was a positive value in kyphosis patients); TLK (the sagittal angle between superior endplate of T10 and inferior endplate of L2); lumbar lordosis (the sagittal angle between superior endplate of L1 and inferior endplate of L5, which was a positive value in lordosis patients) (Fig. 1). These parameters were collected by 2 investigators independently.

### 2.3 Abdominal imaging parameters

Normally, the deformity is evaluated in a standing positon as a standard manner, however, standing radiographs cannot indicate abdominal factors, in addition, patients with degenerative spinal deformities usually do not receive full-length CT. Thus, MRI was obtained using a 1.5-T scanner (Gyroscan Intera; Philips Medical Systems, NL). Axial slices (4 mm) with 1-mm overlap were acquired using a 3-dimensional thick T1-weighted spin-echo axial scan through the vertebral bodies (TR, 5000 ms; TE, 120 ms; FOV, 250 mm; matrix size, 250 × 360). The same MRI scans and image acquisition protocol were applied for supine position.

The calculation of the ACV was performed with equations using the longitudinal (a), transverse (b), and anterior-posterior (c) diameters of the abdominal cavity,^[8]^ where ACV = 0.52 × a × b × c.^[9]^

**Figure 2:**
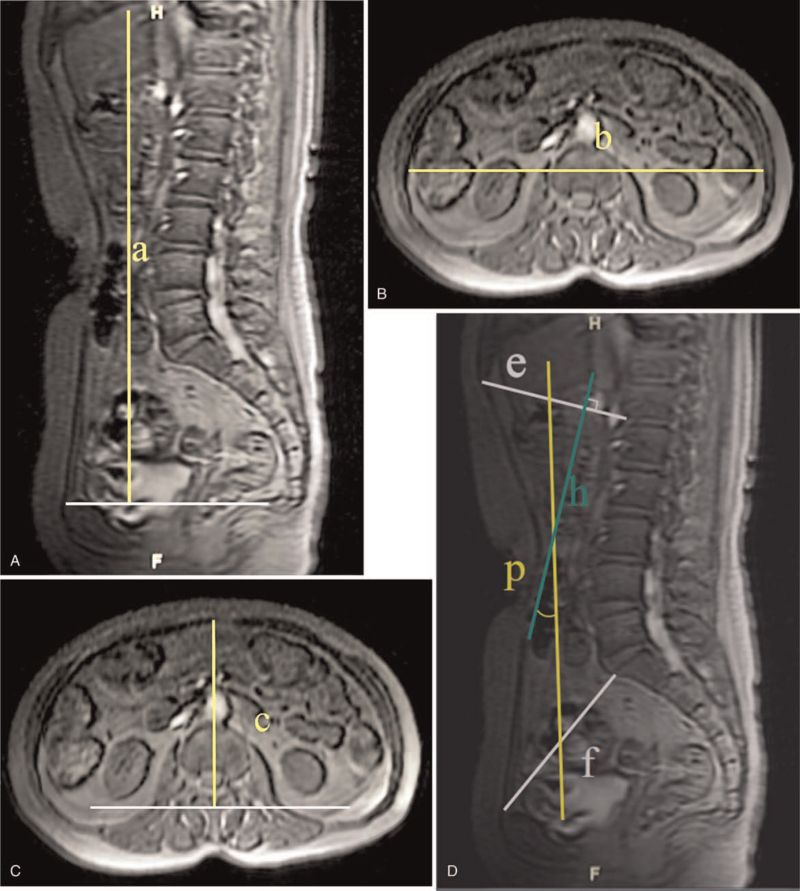
Measurements of ACV and DR on MRI. (A) Longitudinal diameters (a) of the abdominal cavity; (B) Transverse diameters (b) of the abdominal cavity; (C) Anterior-posterior diameters (c) of the abdominal cavity; (D) measurements of DR on MRI ACV = abdominal cavity volume, DR = diaphragm rotation.

The longitudinal diameter of the abdominal cavity was defined as the vertical distance from the diaphragmatic dome to the level of the symphysis pubis. On the axial MRI scan, the transverse diameter of the abdominal cavity was measured at the level of L3. The anterior-posterior diameter of the abdominal cavity was defined as the vertical distance from the apex of the parietal peritoneum of the anterior wall to the horizontal line that passes through the transverse process or the posterior edge of the spinal canal of the L3 vertebrae (Fig. 2).

On the sagittal plane, the diaphragm rotation (DR) was measured^[10]^ (Fig. 2), the DR was defined as the angle between line h and line p. Line e is between the xiphoid process and the anteroinferior edge of T12 and line h is perpendicular to the diaphragm line e. Line f is between the superior edge of the pubis and the anterosuperior corner of the sacrum and line p is between the midpoints of line e and line f. The DR was used to quantify the rotation degree of diaphragm. All the values were measured twice by 2 independent observers and the average was calculated.

### 2.4 Body mass index

Body mass index (BMI) equals weight (kg) divided by the square of height (m^2^).

### 2.5 Statistical analysis

Images were analyzed using PACS client software (Easy Vision IDS5, version 11.4; Philips, Hamburg, Germany). Dichotomous variables were tested by χ^2^ test between the control and DSD groups. The independent sample *t* test was used to compare inter-group parameters. The parameters of DLS group, DK group, and DKS group with DSD were evaluated by analysis of variance. Pearson correlation analysis was used to evaluate the correlation of ACV and DR. Multiple regression model was used to evaluate the influencing factors of ACV and DR. The statistical analysis was performed by SPSS 22.0 (International Business Machines Corporation, Armonk, NY) and statistical significance was defined as *P* < .05.

## 3 Results

Eventually, an overall 95 patients with DSD were recruited and 100 subjects were selected into control group. There were 31 patients in DLS group, 32 patients screened into DK group, and 32 patients screened into DKS group. There was no statistical difference in gender distribution between DSD and control groups (*P* = .96). The age (*P* = .35), BMI, height, and weight were well-matched between the 2 groups (*P* > .05) (Table 1). In DSD group, it was comparable among DLS groups, DK group, and DKS group in terms of gender (*P* = .90), age (*P* = .35), and BMI (*P* = .69) at baseline (*P* > .05).

**Table 1:** Demographic characteristics between DSD and control groups.

	DSD group	Control group	*P*
Cases	95	100	
Gender (M/F)	25/70	26/74	.962
Age (yrs)	68.38 ± 8.42	65.11 ± 5.92	.125
Height (cm)	160.7 ± 8.43	161.72 ± 7.35	.664
Weight (kg)	65.30 ± 9.22	66.61 ± 8.16	.544
BMI (kg/m^2^)	25.16 ± 3.31	25.49 ± 2.94	.698

BMI = body mass index, DSD = degenerative spinal deformity.

Thirty-one cases of DLS with coronal Cobb angle of 28.7° ± 7.7°, 32 cases of DK with TLK of 35.7° ± 9.4°, 32 patients with DSK with coronal Cobb of 31.5° ± 9.9°, and TLK of 26.5° ± 2.6° (Table 2).

**Table 2:** The radiographic parameters on X-ray among 4 groups.

Parameters (°)	Control group	DLS group	DK group	DKS group
Cobb angle	1.6 ± 3.4	28.7 ± 7.7	2.3 ± 2.5	31.5 ± 9.9
TK	23.9 ± 5.6	10.2° ± 2.4	32.7 ± 12.6	28.2 ± 5.6
TLK	3.0 ± 4.5	8.2 ± 4.3	35.7 ± 9.4	26.5 ± 2.6
LL	39.6 ± 10.0	20.6 ± 7.0	42.5 ± 4.4	36.5 ± 7.6

DK = degenerative kyphosis, DKS = degenerative kyphoscoliosis, DLS = degenerative lumbar scoliosis, LL = lumbar lordosis, TK = thoracic kyphosis, TLK = thoracolumbar kyphosis.

**Figure 3:**
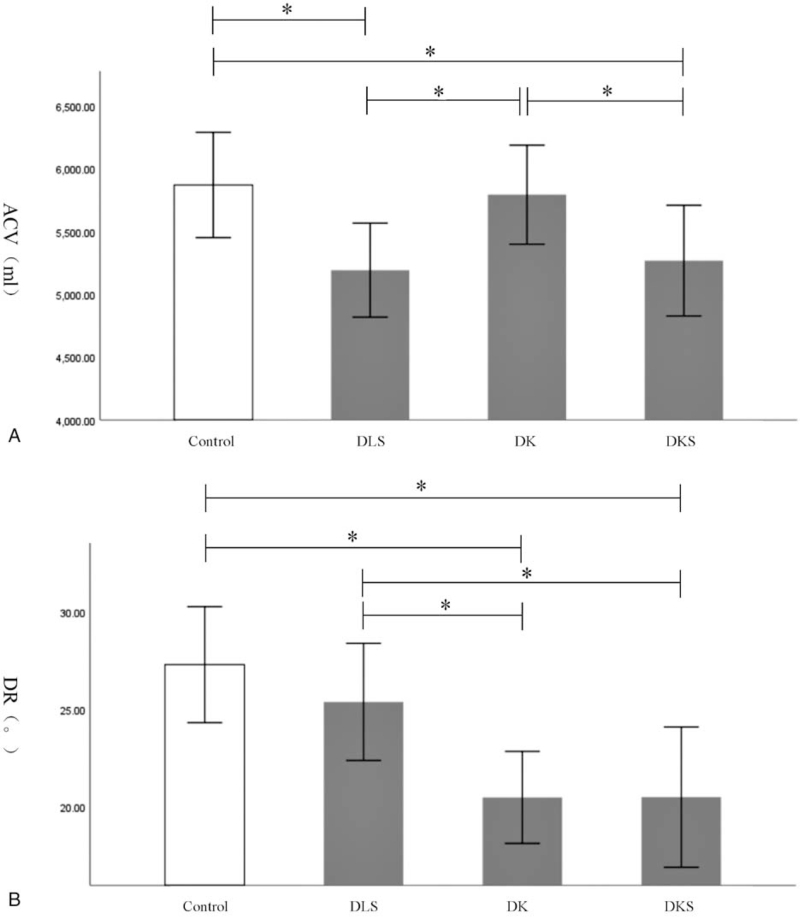
Comparisons on ACV and DR among DLS group, DK group, and DKS group. ACV = abdominal cavity volume, DK = degenerative kyphosis, DKS = degenerative kyphoscoliosis, DLS = degenerative lumbar scoliosis, DR = diaphragm rotation. ^∗^: significance with *P* < .05.

ACV of DLS group and DKS group was significantly lower than control group (*P* < .05) while DR in DK group and DKS group was significantly lower than normal ones (*P* < .05). The inter-group of DSD analysis showed ACV in DLS group and DKS group got decreased than those in DK group (*P* = .032 and *P* = .040, respectively). DR in DK group and DKS group was significantly higher than that in DLS group (*P* = .007 and *P* = .009, respectively) (Table 3 and Fig. 3).

**Table 3:** Comparisons on ACV and DR among 4 groups.

Parameters	Control group	DLS group	DK group	DKS group	*P*
ACV (mL)	5882.60. ± 906.14	5180.20 ± 938.12	5793.84 ± 900.52	5268.26 ± 992.98	.021
a (cm)	33.15 ± 2.47	32.32 ± 2.02	32.94 ± 2.47	31.30 ± 2.14	.040
b (cm)	25.17 ± 1.89	24.62 ± 1.83	24.77 ± 2.12	23.52 ± 2.21	.113
c (cm)	13.79 ± 1.65	12.33 ± 1.47	13.61 ± 1.80	13.72 ± 1.80	.005
DR (°)	27.30 ± 5.97	26.24 ± 6.24	20.50 ± 6.54	20.51 ± 8.10	.032

ACV = abdominal cavity volume, DK = degenerative kyphosis, DKS = degenerative kyphoscoliosis, DLS = degenerative lumbar scoliosis, DR = diaphragm rotation. a: longitudinal diameters of the abdominal cavity; b: transverse diameters of the abdominal cavity; c: anterior-posterior diameters of the abdominal cavity.

The ACV is positively correlated to the height (r = 0.291, *P* = .013) and weight (r = 0.700, *P* = .001) while negatively to Cobb angle (r = –0.562, *P* = .001). When ACV is seen as dependent variable and the correlated parameters are included, it shows weight and Cobb angle are independent influencing factors for ACV with ACV (mL) = 0.67 × weight – 0.19 × Cobb angle + 2231.8. The DR is negatively to TLK (r = –0.353, *P* = .003) and TLK is independent influencing factor with DR (°) = 25.82 – 0.42 × TLK. The shortest distance between xiphoid process and spine = 0.03 × TLK + 0.08 × body weight + 4.95 (R2 = 0.32, *P* < .05) (Table 4).

**Table 4:** Independent influencing factors for ACV and DR.

		Unstandardized		Standardized		
Dependent variables	Coefficient	B	SE	Beta	t	*P* value
ACV	Constant	2231.821	84.561		9.285	.022
	Weight	58.278	9.694	0.665	7.683	.000
	Cobb	−27.173	1.406	−0.191	−2.503	.014
	Height	4.196	6.007	0.032	−0.381	.804
DR	Constant	25.823	1.238		22.111	.000
	TLK	−0.120	0.046	−0.418	−4.369	.000

ACV = abdominal cavity volume, DR = diaphragm rotation, TLK = thoracolumbar kyphosis.

## 4 Discussion

Prior studies have noted the importance of the effect of spinal deformity on body function, severe AS kyphosis can lead to respiratory dysfunction,^[11]^ which is mainly due to interstitial lung disease or thoracic wall abnormalities.^[12]^ Kyphosis of AS will also oppress the abdominal cavity, resulting in a decrease in ACV.^[8]^ After orthopedic surgery, weight, gastrointestinal function, and defecation frequency have changed significantly.^[13]^ AIS can lead to pulmonary function limitation, and there was a negative correlation between vital capacity, maximum ventilation, arterial oxygen saturation, and the degree of scoliosis.^[14]^ DSD is a 3-dimensional rotational deformity that occurs mainly in the thoracolumbar segment,^[15]^ and may lead to changes of ACV. However, there has been no detailed investigation the effect of DSD on ACV or DR of diaphragm related to the change of abdominal cavity. In this work, a comparison and analysis of the imaging changes of abdominal cavity of degenerative scoliosis, degenerative TLK, and degenerative scoliosis were reported for the first time to our knowledge. The ACV of patients with coronal plane deformities such as DLS and DKS decreased significantly compared with the control group. The rotation angle of diaphragm in patients with sagittal kyphosis is also significantly smaller.

A number of recent studies have investigated the relationship between trunk cavity volume and spinal deformity. Ji et al^[8]^ studied 29 patients with AS and found that the ACV of them (average GK 76°) increased by 652 mL after orthopedic surgery, indicate that kyphosis can lead to ACV decrease. Also, in this study, they found the reason of the ACV's change is mainly due to the decrease of the longitudinal diameters (a) of the abdominal cavity. In AIS patients, Newton et al^[16]^ studied 631 patients with AIS. Imaging analysis showed that thoracic rotation caused by AIS scoliosis reduced thoracic volume.

Different from Ji et al's^[8]^ study on kyphosis of AS, in our study, simple sagittal plane deformities such as degenerative TLK have no obvious abnormality in ACV. On the one hand, unlike AS, degenerative TLK only exist in thoracolumbar segment, and have limited impact on longitudinal diameter (a), and DK on the other hand has a greater range of motion and less degree of deformity than AS, body can compensate for this change to some extent through the thoracic vertebrae and pelvis.^[17]^ It is worthy of attention that the reduction of abdominal volume caused by degenerative scoliosis is mainly due to the decrease of the anterior-posterior diameter of the abdominal cavity, which may be caused by the rotation of the vertebral body caused by scoliosis deformity. Liljenqvist et al^[18]^ found that the rotation of the vertebral body can cause the posterior peritoneum to move to the ventral side. Therefore, the rotation of the vertebral body caused by scoliosis will lead to a decrease in the anterior-posterior diameter (c) of the abdominal cavity. At the same time, the abdominal volume of DSK patients was also lower than that of the control group, their anterior-posterior diameter (c) was significantly reduced due to the rotation of the vertebral body, and the longitude diameter (a) the abdominal cavity was also reduced to a certain extent.

**Figure 4:**
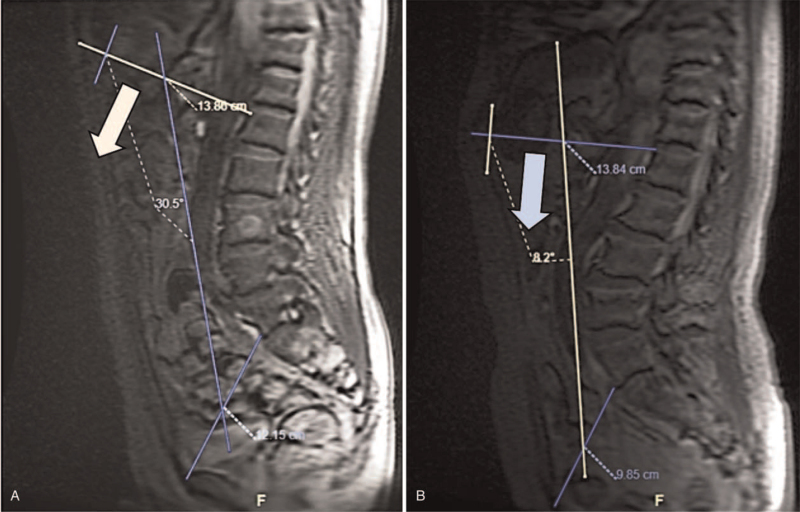
DR and diaphragm movement from control group and DK group. (A) A case from control group. A 64 year-old female, with the height of 163 cm and weight of 66.2 kg/m^2^, TLK of 3.0° and DR of 30.5°; (B) A case from DK group. A 65 year-old female, with the height of 165 cm and weight of 65.8 kg/m^2^, TLK of 34.0° and DR of 8.2°. DK = degenerative kyphosis, DR = diaphragm rotation, TLK = thoracolumbar kyphosis.

As an important respiratory muscle, the diaphragm constitutes abdominal breathing.^[19]^ Deformity of the spine will cause the rotation of the diaphragm, which in turn affects the activity of the diaphragm.^[20]^ Di Bari et al^[4]^ conducted a comparative analysis of 130 patients with DK and 200 controls. It was found that forced vital capacity (FVC), forced expiratory volume in first second, and forced expiratory volume in first second/FVC were significantly decreased in patients with DK. Another study^[10]^ analyzed the relationship between DR and pulmonary function in AS. This study found that there was a correlation between DR angle and vital capacity, FVC, expiratory reserve volume, inspiratory reserve volume, and peak expiratory flow. According to the theory of Duomarco,^[21]^ the contraction and relaxation of the diaphragm during breathing is related to the extension of the anterior abdominal wall. When exhaling hard, the abdominal muscle plays an important role. Therefore, the movement of the diaphragm in patients with kyphosis is changed to top to bottom (Fig. 4), which is limited by viscera and posterior spine. This is why expiratory reserve volume and inspiratory reserve volume are significantly correlated with the rotation angle of the diaphragm.

In this study, the DR was measured in patients with DSD. It was found that compared with the control group, the DR of the DK group and the DSK group was significantly smaller, this difference may reduce the respiratory function reserve and the operational tolerance of DK patients.^[21]^ Moreover, our study measures the diameters through CT and MRI, the kyphosis were extended during examination in supine position, and the measured DR angle was less severe, which means that patients with DK may have more severe DR. Thus, for patients with severe TLK, routine pulmonary function test is needed to find potential pulmonary dysfunction.

The reported incidence of dyspepsia in people aged 65 and above is as high as 24.4%.^[22]^ For patients with degenerative scoliosis, long-term bed rest due to discomfort has affected gastrointestinal peristalsis. On this basis, the reduction of abdominal volume caused by scoliosis may further affect on digestive function and prolong the time of rehabilitation after operation.^[23]^ Conversely, for patients with abnormal digestive function, it is necessary to consider the impact on scoliosis and make a reasonable operation. In addition, patients with degenerative scoliosis usually undergo posterior surgery, when the patient is in the prone position, the abdominal volume is further compressed, an increase in abdominal pressure may increase the amount of blood loss during the operation. At the same time, the position of the abdominal aorta may change after visceral compression, increasing the risk of damage during pedicle screw placement.^[24]^

This study has the following limitations. First of all, this study compares the pre-operative imaging data of different patients instead of comparing the difference before and after surgery, and there are inherent limitations in attempts to assess 3-dimensional structure from 2-dimensional images. Secondly, patients with DSD usually do not undergo complicated examinations unless the condition is severe, this study lacks the analysis of patients’ pulmonary function and digestive function, further research should be undertaken to investigate the function measurement through the respiratory function test, gastrointestinal Nepean Dyspepsia Index, Leeds dyspepsia questionnaire, to further study the relationship between parameters and function.

## 5 Conclusion

DLS can cause the decrease of ACV and the degree of scoliosis is negatively correlated to ACV but not to DR. Patients with severe DLS need orthopedics surgery to improve abdominal volume. DK will not cause changes of ACV but is related to the degree of kyphosis. Therefore, for patients with severe DK, pulmonary function tests are required to rule out potential abnormalities. DKS will impact both ACV and DR and it is necessary to be paid attention to even the function of thoracic and abdominal cavity for this group.

## Author contributions

**Conceptualization:** Chen Guo, Yan Liang.

**Data curation:** Chen Guo, Shuai Xu.

**Formal analysis:** Chen Guo, Shuai Xu.

**Funding acquisition:** Chen Guo.

**Investigation:** Chen Guo.

**Methodology:** Chen Guo, Yan Liang.

**Project administration:** Chen Guo, Yan Liang.

**Resources:** Chen Guo, Yan Liang, Zhenqi Zhu.

**Software:** Chen Guo, Fanqi Meng.

**Supervision:** Chen Guo.

**Validation:** Chen Guo, Fanqi Meng.

**Visualization:** Chen Guo, Fanqi Meng.

**Writing – original draft:** Chen Guo.

**Writing – review & editing:** Zhenqi Zhu, Haiying Liu.
